# Supplementary material for: Cysteine Sulfoxides Enhance Steroid Hormone Production via Activation of the Protein Kinase A Pathway in Testis-Derived I-10 Tumor Cells
Source: Molecules. 2020 Oct 14;25(20):4694. doi: 10.3390/molecules25204694 (PMC7587355; doi:10.3390/molecules25204694)
Supplement: Supplementary file 1 [file molecules-25-04694-s001.pdf]

molecules

Supplementary Material

Cysteine Sulfoxides Enhance Steroid Hormone Production via Activation of the Protein Kinase A Pathway in Testis-Derived I-10 Tumor Cells

Yuya Nakayama [1,2,3,*,†], Hsin-Jung Ho [3,4,†], Miki Yamagishi [2], Hiroyuki Ikemoto [1], Michio Komai [3] and Hitoshi Shirakawa [3]

[1] Health Care Research Center, Nisshin Pharma Inc., Saitama 356-8511, Japan; ikemoto.hiroyuki@nisshin.com
[2] Research Center for Basic Science, Research and Development, Quality Assurance Division, Nisshin Seifun Group, Inc., Saitama 356-8511 Japan; yamagishi.miki@nisshin.com
[3] Laboratory of Nutrition, Graduate School of Agricultural Science, Tohoku University, Sendai 980-8572, Japan; hsinjung@hs.hokudai.ac.jp (H.-J.H.); mkomai@m.tohoku.ac.jp (M.K.); shirakah@tohoku.ac.jp (H.S.)
[4] Faculty of Health Sciences, Hokkaido University, Sapporo 060-0812, Japan
* Correspondence: nakayama.yuya@nisshin.com; Tel.: +81-49-267-3928; Fax: +81-49-266-2749
† These authors contributed equally to this work.

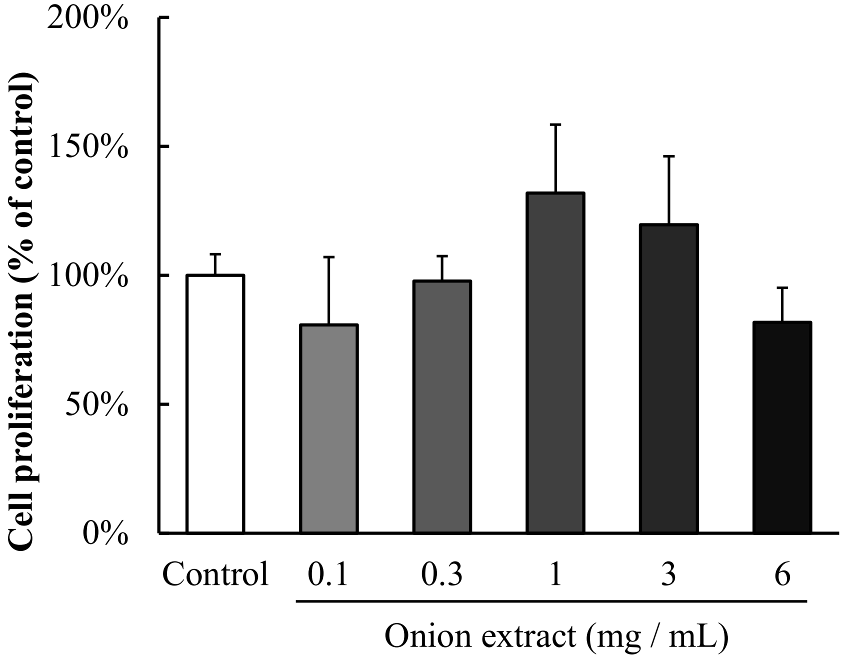

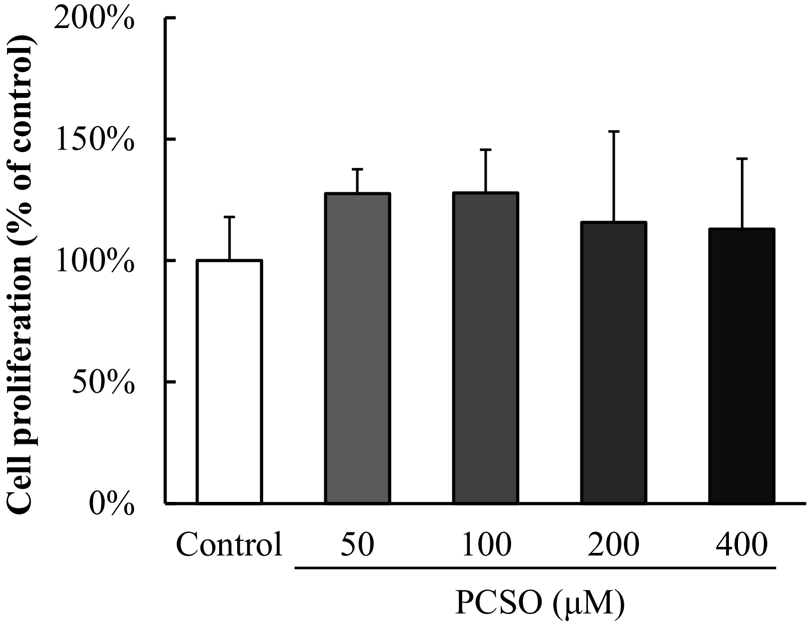
200%
150%
100%
50%
0%
Cell proliferation (% of control)
Control
50
100
200
400
PCSO (μM)

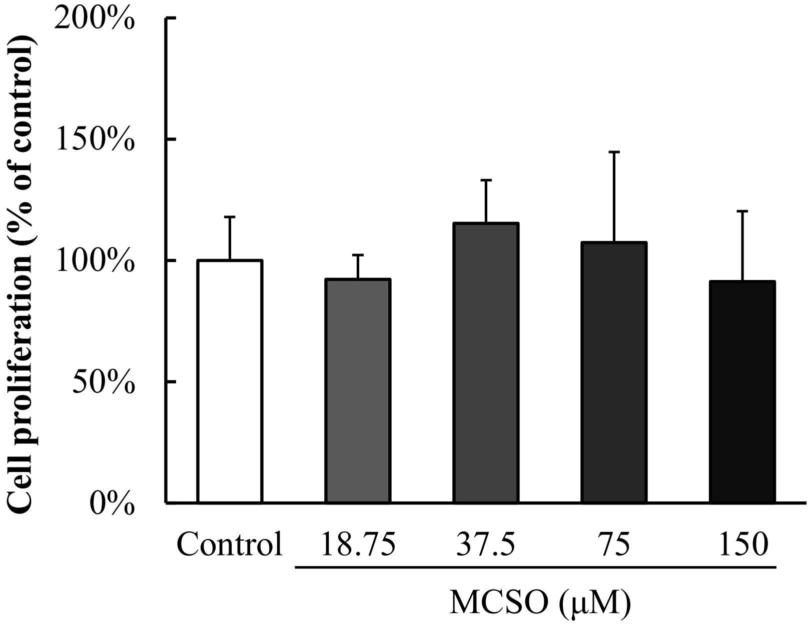
200%
150%
100%
50%
0%
Cell proliferation (% of control)
Control
18.75
37.5
75
150
MCSO (μM)

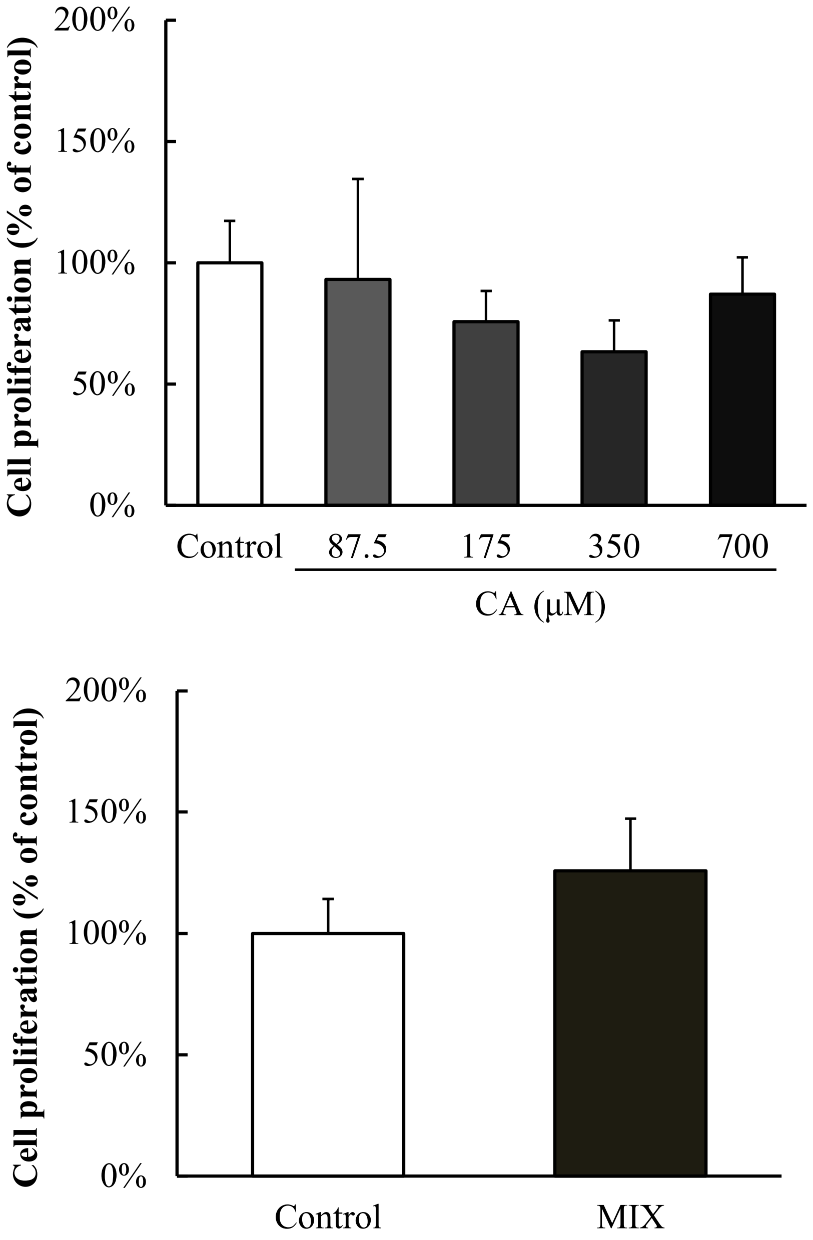

Figure S1. Effects of onion extract (PCSO, CA, MCSO, and MIX [PCSO 400 μM, MCSO 150 μM, and CA 700 μM]) on cytotoxicity in I-10 cells. Data are presented as mean ± standard deviation (n = 5). PCSO: propenyl-l-cysteine sulfoxide; MCSO: S-methyl-L-cysteine sulfoxide; CA: cycloalliin.

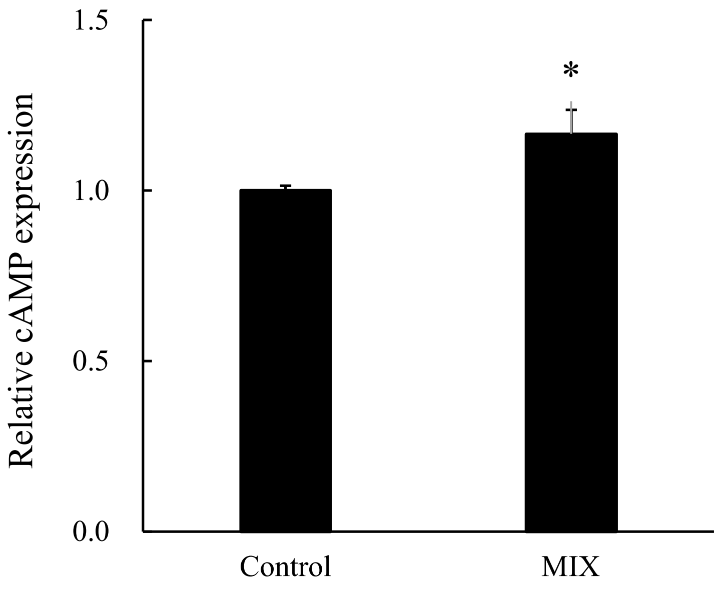

Figure S2. The enhancement of cAMP production by MIX (PCSO 400 μM, MCSO 150 μM, and CA 700 μM) in I-10 cells. Data are presented as mean ± standard deviation (n = 3). *$p < 0.05$ vs. control. PCSO: propenyl-l-cysteine sulfoxide; MCSO: S-methyl-L-cysteine sulfoxide; CA: cycloalliin; cAMP: cyclic adenosine monophosphate.
